# Characterization of the Cryptic AV3 Promoter of Ageratum Yellow Vein Virus in Prokaryotic and Eukaryotic Systems

**DOI:** 10.1371/journal.pone.0108608

**Published:** 2014-09-30

**Authors:** Wei-Chen Wang, Chia-Ying Wu, Yi-Chin Lai, Na-Sheng Lin, Yau-Heiu Hsu, Chung-Chi Hu

**Affiliations:** 1 Graduate Institute of Biotechnology, National Chung Hsing University, Taichung, Taiwan; 2 Institute of Plant and Microbial Biology, Academia Sinica, Nankang, Taipei, Taiwan; Institute of Infectious Disease and Molecular Medicine, South Africa

## Abstract

A cryptic prokaryotic promoter, designated AV3 promoter, has been previously identified in certain begomovirus genus, including ageratum yellow vein virus isolate NT (AYVV-NT). In this study, we demonstrated that the core nucleotides in the putative −10 and −35 boxes are necessary but not sufficient for promoter activity in *Escherichia coli*, and showed that AYVV-NT AV3 promoter could specifically interact with single-stranded DNA-binding protein and sigma 70 of *E. coli* involved in transcription. Several AYVV-NT-encoded proteins were found to increase the activity of AV3 promoter. The transcription start sites downstream to AV3 promoter were mapped to nucleotide positions 803 or 805 in *E. coli*, and 856 in *Nicotiana benthamiana*. The eukaryotic activity of AV3 promoter and the translatability of a short downstream open reading frame were further confirmed by using a green fluorescent protein reporter construct in yeast (*Saccharomyces cerevisiae*) cells. These results suggested that AV3 promoter might be a remnant of evolution that retained cryptic activity at present.

## Introduction

The members of the family *Geminiviridae* are characterized by their single-stranded circular DNA genomes and the geminate-shaped virus particles. Geminiviruses are divided into seven genera, *Mastrevirus*, *Curtovirus*, *Begomovirus*, *Topocuvirus*, *Becurtovirus*, *Eragrovirus*, and *Turncurtovirus* based on host range, vector specificities and genome organization [1]–[3]. Although geminiviruses are plant viruses, they have been suggested to have prokaryotic origins [4]–[7]; based on several prokaryotic features, including the rolling-circle replication mechanism analogous to some bacteriophages and eubacterial plasmids [8], [9], and the similarities in conserved motifs between geminivirus-encoded replication-associated protein (Rep) and the replication initiator proteins of plasmids of pMV158 family of bacteria [6].

Previous studies have demonstrated that some geminiviruses could produce various DNA forms indicative of replication processes and express certain viral genes in *Agrobacterium tumefaciens* and *Escherichia coli* [10], [11]. We have also demonstrated that unit-length single-stranded circular DNA of *Ageratum yellow vein virus* (AYVV) in the genus *Begomovirus* can be generated in *E. coli* harboring AYVV genome with only a single origin of replication (*ori*) cloned in phage M13 vector [12]. These observations suggested that certain regulatory sequences involved in viral gene expression might be active in both prokaryotic and eukaryotic systems.

Recently, a novel cryptic prokaryotic promoter, designated AV3 promoter, was identified at positions near the 3′-terminus of coat protein (CP) open reading frame (ORF) in some monopartite begomoviruses genomes [13]. The AV3 promoter activity is similar to the well-characterized *E. coli* constitutive promoter of ribosomal RNA, *rrn*B P1 promoter [14]. The presence of a downstream prokaryotic ribosome binding site (RBS), a proper spacer, and the translatability in *E. coli* of a small ORF downstream to AV3 promoter were also confirmed. These findings further supported the prokaryotic origins of geminiviruses, and revealed that certain prokaryotic features in the geminivirus genomes are still retained and possibly functional in their infection cycles. However, these observations also raised certain key questions about the regulation and activity of AV3 promoter in prokaryotic and possibly in eukaryotic systems: Whether the putative core motifs (−10 and −35 boxes) found in AV3 promoter are truly functional? What are the factors interacting with AV3 promoter in *E. coli*? Whether the AV3 promoter is also active in eukaryotic systems? And if so, whether the small ORF downstream to AV3 promoter is translatable in the eukaryotic system?

To address these questions, we further characterized the regulatory features of ageratum yellow vein virus isolate NT (AYVV-NT) AV3 promoter in this study. Several lines of evidence were presented confirming that AV3 promoter of AYVV-NT is active in *E. coli* and suggested that AV3 promoter might exhibit a level of activity in plant and yeast cells.

## Materials and Methods

### Viruses

AYVV-NT and tomato leaf curl virus (TLCV), which is another monopartite begomovirus, respective full-length clones, pAYVVNT and pTLCV, have been described previously [13], [15]. The nucleotide sequence of AYVV-NT genome has been deposited in GenBank under the GenBank index (GI) number: 149193093.

### Plasmids

To generate the AV3 promoter mutants, 5′-phosphorylated primer pairs listed in Table 1 (purpose A) were used to construct pAYAV3PM and pTLAV3PM using pAYVVNT and pTLCV as the templates by inverse polymerase chain reaction (IPCR). The AV3 promoter-corresponding regions of AYVV-NT and TLCV mutants were amplified by PCR using primer pairs listed in Table 1 (purpose B) using pAYAV3PM and pTLAV3PM as templates. The PCR products were cloned into the pGlow-TOPO vector (Invitrogen, Life Technologies, Carlsbad, CA, USA), which harbors the Cycle 3 green fluorescence protein (GFP) as a reporter, to create pAY-M and pTL-M. For infectivity assays, infectious construct was prepared essentially as described previously [15]. The plasmid pAYAV3PM was used to generate tandem dimer of full-length genome of AVVV-NT mutant. The resulting products were cloned into the pBin19 vector to create pAYAV3PM-DI.

**Table 1:** Primers used in this study.

Primer name	Purpose^a^	Oligonucleotide sequence (5′→3′)^b^	Position^c^
AYAV3PM-F	A	5Phos-TAGGCCCAGCACTGCCACCATCAAGAATGATCTTC	AY777-811
AYAV3PM-R	A	5Phos-TTATCGTACATGTTAAACACCTGACCAAAATCC	AY776-744
TLAV3PM-F	A	5Phos-TCAAGAATGATAATAGAGATAGGTTTCAAG	TL797-826
TLAV3PM-R	A	5Phos-TAGTAGCAGTACTAGGCTCATTATCATAC	TL796-768
AY762F-771C	B	TAACATGTACGATAATGAGCCCA	AY762-784
AY869R	B, F	CCATATCTATATC**TCCT**TGCATATTGACCC	AY869-858
TL762F	B	TAATATGTATGATAATG	TL762-779
TL869R	B	CCATATCTATATC**TCCT**TGCATACTGACCGCCA	TL869-855
SSB-NcoIF	C	AGCCATGGCTATGGCCAGCAGAGGC	NA
SSB-XhoIR	C	AGCTCGAGGAACGGAATGTCATCATC	NA
pBT1634SacIF	D	AGGAGCTCAAAAAGAAACCATTAACAC	NA
pBT1634SacIR	D	GTGAGCTCCATACGCTGTTTCCTG	NA
AV1-SacIF	D	CTGAGCTCATGTGGGATCCTCTTT	AY132-147
AV1-XhoIR	D	AGCTCGAGTCAGGGGTTCTGTACA	AY482-467
CP-SacIF	D	CTGAGCTCATGTCGAAGCGTCCCG	AY292-307
CP-XhoIR	D	AGCTCGAGTTAATTCTGAACAGAA	AY1065-1050
Rep-SacIF	D	CTGAGCTCATGGCTCCTCCAAGAC	AY2602-2587
Rep-XhoIR	D	AGCTCGAGCTACGCCTGCGAACTG	AY1520-1535
TrAP-SacIF	D	TCGAGCTCATGCGGAATTCGTCACC	AY1614-1598
TrAP-XhoIR	D	TCCTCGAGCTAAATACCCTCAAGAA	AY1207-1223
REn-SacIF	D	CTGAGCTCATGGATTTTCGCACAG	AY1466-1451
REn-XhoIR	D	AGCTCGAGTTAATAAAGATTGAAT	AY1062-1077
C4-SacIF	D	CTGAGCTCATGGGAGCCCTCATCT	AY2445-2430
C4-XhoIR	D	AGCTCGAGTTACATTAAGAGCCTC	AY2155-2170
C3GFP-KpnIF	E	AGGGTACCCATGGCTAGCAAAG	NA
C3GFP-KpnIR	E	AGGGTACCTTATTTGTAGAGCTC	NA
AY762-SpeIF	E	AGACTAGTTAACATGTATGATAATG	AY762-778
Biotin-AY615F	F	5Bio-GAGATTCTGTGTTAAGTC	AY615-632
Biotin-rrnbp1F	F	5Bio-CCGATGCGAATATTGCCT	NA
rrnbp1R	F	CTGCCGGAGTTCTCAGGA	NA
AY1022R	G	GTAGCATACACAGGATTACTGGCATGAG	AY1022-995
UPM	G	Long (0.4 µM)-CTAATACGACTCACTATAGGGCAAGCAGTGGTATCAACGCAGAGTShort (2 µM)- CTAATACGACTCACTATAGGGC	NA
AY950R	G	TACTTAGCAGCTTCTTGATG	AY950-931
AY868F	G	GCTTCTAAGGAACAGGCGTTGGTGAAG	AY868-894

a The main purpose for the primers. A, for mutating the AV3 promoter of the AYVV-NT and TLCV genomes; B, for amplifying the corresponding regions of AV3 promoter from virus genome; C, for construction of the *E. coli* His_6_-tagged SSB protein expression vector; D, for detection of the effects of virus-encoded proteins on the AV3 promoter activity; E, for verifying the AV3 promoter activity and translatability in yeast. F, for identifying the transcription factor interacts with AV3 promoter; G, for 5′ RACE and 3′ RACE studies.

b The letters in bold indicates the position of the RBS (in the complementary sense of AGGA), and the start codon (ATG) is underlined.

c The relative position of the primer sequences in the respective viral genome. AY, AYVV-NT; TL, TLCV; NA, not applicable.

For producing the His_6_-tagged single-stranded DNA-binding (SSB) proteins, the plasmid pET21d-SSB was generated by inserting the *E. coli* SSB gene sequence (GI: 557274221) using the primer pair listed in Table 1 (purpose C). The His_6_-tagged SSB were over-expressed in *E. coli* BL21 (DE3) and purified by Ni-NTA purification system (Invitrogen, Life Technologies, Carlsbad, CA, USA).

A series of expression vectors for AYVV-NT-encoded proteins were generated based on the modified pBT plasmid (Agilent Technologies Inc., Santa Clara, CA, USA). The pBT vector was mutated to generate pBT-1634SacI by in-frame inserting a *Sac*I site downstream to the start codon of the lambda-cI gene, using IPCR with the primer pair pBT1634SacIF and pBT1634SacIR (Table 1). Each of AYVV-NT-encoded genes was amplified using specific primer pairs (Table 1, purpose D), and then inserted into the *Sac*I and *Xho*I digested pBT-1634SacI vector to create pBT-AV1, pBT-CP, pBT-Rep, pBT-TrAP, pBT-REn and pBT-C4, respectively. The lambda-cI protein ORF is replaced by the respective ORFs of AYVV-NT-encoded proteins in the cloning process.

For yeast assays, the pYES2/NT-C vector (Invitrogen, Life Technologies, Carlsbad, CA, USA) was modified to replace the original GAL1 promoter with two different AYVV-NT fragments comprising the same AV3 promoter region plus two downstream ORFs fused to the Cycle 3 GFP gene. Two plasmids described in our previous study, pGP762-889GFP and pGP762-1062GFP [13], were used as templates. The primer pair AY762-SpeIF and C3GFP-KpnIR (Table 1) was used to amplify the corresponding regions with GFP fusion fragments. The PCR products were cloned into the *Spe*I and *Kpn*I digested pYES2/NT-C vector to generate the pY762-889GFP and pY762-1062GFP. A positive control, pYES2-cycle3GFP, was also generated (using primer pair C3GFP-KpnIF plus C3GFP-KpnIR), in which the Cycle3 GFP gene was inserted under the control of the original GAL1 promoter.

### Promoter activity assays in *E. coli*

GFP-based promoter activity assays were performed and analyzed as described previously [16] with minor modifications. Individual constructs, pGlow-TOPO, pGP762-869 [13] (abbreviated as pAT-WT in this study), pAY-M, pGP762-869TLCV [13] (abbreviated as pTL-WT), and pTL-M, were used to transformed TOP10 *E. coli*. Bacteria harboring each construct were cultivated in LB broth containing 100 µg of ampicillin ml^−1^ at 37°C for 16 h. The cultures were then diluted 200-fold and incubated at 37°C for 4 h to reach the mid-log phase. Aliquots of 150 µl liquid cultures were loaded into a 96-well plate with three duplicates, and the fluorescence were measured by using an FLx800 Multi-Detection Micro-plate Reader (BioTek Instruments, Winooski, VT, USA) at an excitation wavelength of 400 nm and an emission wavelength of 508 nm, with a sensitivity setting of 60. The optical densities of each bacterial culture at 600 nm (OD_600_) were measured by using a SpectraMax M2 microplate reader (Molecular Devices, Sunnyvale, CA, USA). Cultures were allowed to continue growing for an additional 1 h, and the GFP fluorescence and OD_600_ were measured again. The promoter activity (indirectly indicated as GFP synthesis rate per cell) of each samples were calculated with three biological duplicates as described by Davis et al. (2011) as follows: the changes in fluorescence between the two readings was divided by the average of OD_600_, followed by correcting for background auto-fluorescence by subtracting the per cell synthesis rate of the negative control (vector only).

### Pull-down assay and western blot analysis

Overnight liquid cultures (1 ml each) of *E. coli* DH5α were harvested and resuspended in 500 µl PBS buffer, followed by sonication with 10 s pulse/5 s pause for 10 min. The products were then incubated with 10 µg of biotin-labelled AV3 promoter or *rrn*B P1 promoter, generated by PCR using specific primers (Table 1, purpose F), at RT for 45 min, in the presence of rifampicin (1 µg ml^−1^) to inhibit the extension of transcription [17]. A reaction without promoter fragments was used as a negative control. The reactions were then incubated with streptavidin magnetic beads (Millipore, Temecula, CA, USA) at RT for 1 h. The bound and flow-through fractions were collected and analyzed through a 12.5% polyacrylamide gel containing 1% SDS (SDS-PAGE).

The proteins in the gel were subsequently visualized by silver staining or analyzed by western blot assay with specific antibodies. In silver staining, the protein bands of interest were carefully sliced from the gel and subjected to protein identification analysis by matrix-assisted laser desorption/ionization time-of-flight mass spectrometry (MALDI-TOF MS). For western blot assay, the proteins were subsequently transferred to the PVDF membrane, and detected by using a monoclonal antibody against *E. coli* RNA polymerase sigma 70 antibody (2G10) (Thermo Scientific, Rockford, IL, USA), followed by a rabbit anti-mouse IgG AP-conjugated antibody (SIGMA-Aldrich, St. Louis, MO, USA).

### South-western blot assay

Aliquots of 20 or 200 ng PCR fragments corresponding to the indicated promoters were analyzed by electrophoresis through a 1% agarose gel, followed by staining with ethidium bromide (EtBr), or by South-western blot assay. For South-western blot, the PCR fragments in agarose gels were transferred to nitrocellulose (NC) membranes and incubated with 100 µg purified His_6_-tagged SSB proteins at RT for 1 h. The bound SSB proteins on the PCR fragments on NC membranes were detected by a monoclonal antibody against histidine tag (AbD Serotec, Kidlington, Oxford, UK), and followed a rabbit anti-mouse IgG AP-conjugated antibody.

### Co-expression of AYVV-NT-encoded proteins with AV3 and *rrn*B P1 promoter

Each of the plasmids pBT-1634SacI, pBT-AV1, pBT-CP, pBT-Rep, pBT-TrAP, pBT-Ren, and pBT-C4 was co-transformed with the pAY-WT or p*rrn*B-P1 [13], in which the *rrn*B P1 promoter was cloned into the GFP-reporter vector pGlow-TOPO, into the *E. coli* XLI-BLUE MRF' strain. Individual colonies of each treatment were selected and cultivated at 30°C overnight. The overnight cultures were diluted 200-fold and incubated for an additional 7 h, followed by the addition of 100 µM isopropyl-1-thio-β-d-galactopyranoside (IPTG) into the medium to induce protein expression for 1.5 h. The promoter activities were analyzed with three replicates as described above, and the relative promoter activity was normalized to the GFP synthesis rate per cell of *E. coli* cells co-transformed with the respective reporter construct and pBT-1634SacI, which expressed a non-viral protein, lambda-cI.

### 5′ and 3′ rapid amplification of cDNA ends (RACE)

The plasmid pAY1-7 [15] was transformed into *E. coli* DH5α cells. The total nucleic acids were extracted and treated with RQI DNase (Promega, Madison, WI, USA) at 37°C for 30 min, and subsequently used as the templates for 5′ RACE, using a SMARTer RACE cDNA amplification kit (Clontech Laboratories Inc., View, CA, USA). The primer AY1022R (Table 1) was used for synthesis of the first strand cDNAs, which were subjected to amplification with primer pair UPM (Clontech Laboratories Inc., View, CA, USA) and AY950R (Table 1). The PCR products were subsequently cloned into the yT&A vector (Yeastern Biotech Co., Taipei, Taiwan) and sequenced.

In *Nicotiana benthamiana*, the 28-day-old seedlings were agro-infiltrated with the pAY1-7 or pAYAV3PM-DI infectious constructs. Total nucleic acids were extracted from the newly emerged leaves at 14 day-post-inoculation (dpi), and subjected to 5′ RACE as described above. For 3′ RACE, the primer AY868F (Table 1) was used for the synthesis of the first strand cDNAs, followed by PCR amplification with the primer pair UPM plus AY868F. The positions of the termini were analyzed as described above.

### Promoter activity assay in yeast

The plasmids pYES2/NT-C, pY762-889GFP, pY762-1062GFP and pYES2-cycle3GFP were transformed individually to yeast INVSc1 strain (Invitrogen, Life Technologies, Carlsbad, CA, USA). Individual colonies were selected and cultivated in SC-U medium containing 2% dextrose at 30°C for 16 h. The overnight cultures were pelleted to remove the medium and resuspended in SC-U induction medium containing 2% galactose to an OD_600_ of 0.5 and incubated for additional 4 h. The promoter activities were determined by measuring the GFP fluorescence and the OD_600_ of individual constructs at 0 h and 4 h, with four replicates, as described above.

For western blot assays, individual colonies of each plasmid were selected and cultivated in 1 ml SC-U medium containing 2% dextrose at 30°C for 20 h. The overnight cultures were pelleted and resuspended in 100 µl 1× SDS loading dye and boiled at 100°C for 10 min, followed by SDS-PAGE and transferred to PVDF membrane. The GFP proteins in the samples were detected by using a GFP monoclonal antibody (SIGMA-Aldrich, St. Louis, MO, USA), followed by a rabbit anti-mouse IgG AP-conjugated antibody. Transiently expressed GFP in *N. benthamiana* at 3 dpi was used as a positive and protein size control, in which the GFP gene was driven by the CaMV 35S promoter.

## Results

### The putative core motifs of AV3 promoter are necessary but not sufficient for transcription activation in *E. coli*

**Figure 1:**
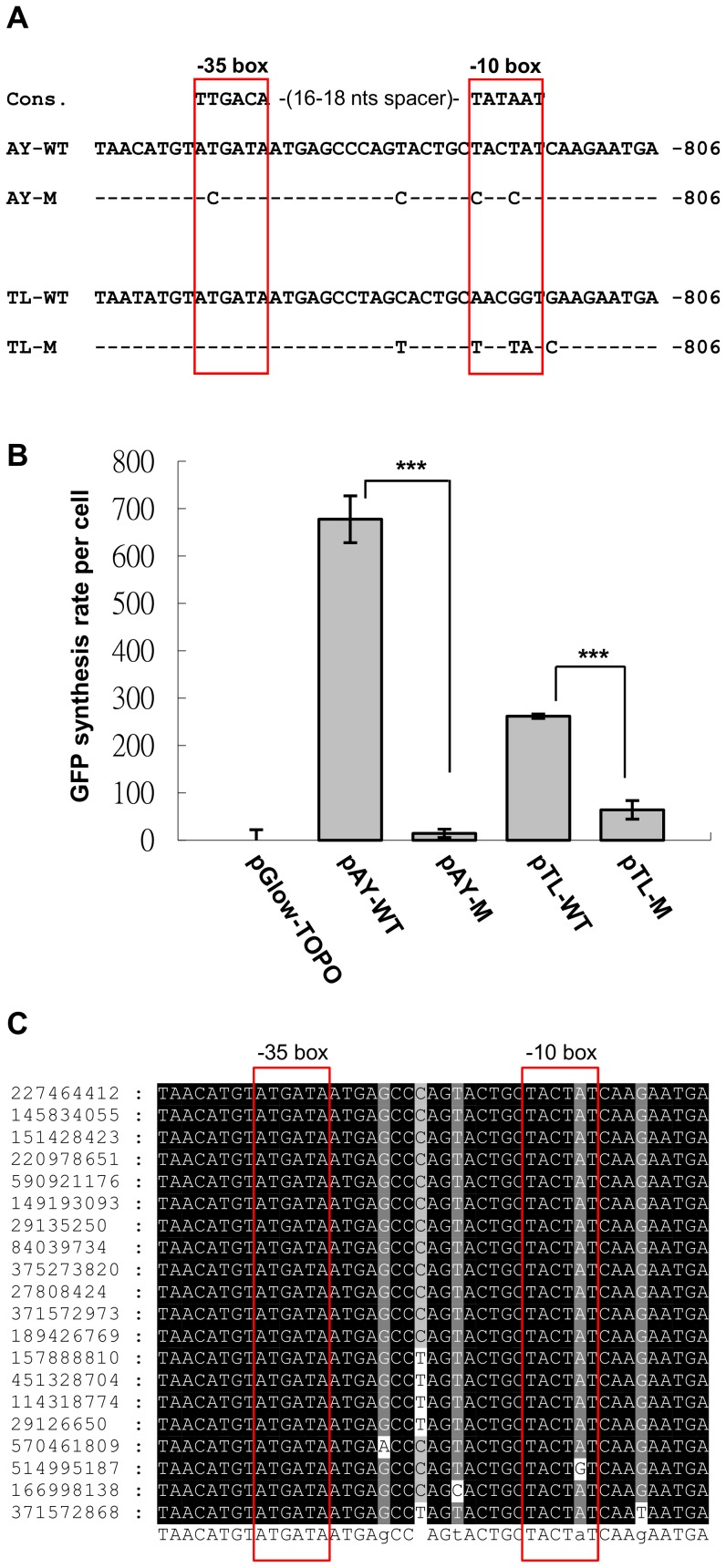
Comparison of the strengths of the wild type and the mutant AV3 promoter. (A) Comparison of nucleotide sequences between the AV3 promoter-corresponding regions in AYVV-NT (AY-WT) and AYVV mutant (AY-M), and those in TLCV (TL-WT) and TLCV mutant (TL-M). The nucleotide positions are indicated by numbers on the right, and the identical nucleotides are indicated by a dash. The canonical sequence of prokaryotic *cis*-element −35/−10 regions (in red boxes) are shown above the alignment. (B) AV3 promoter activity assay. The promoter activities, indirectly expressed as the GFP synthesis rate per cell are shown in the chart. The statistical significance of difference between selected pairs was determined by Student's *t*-test. (***, *p*<0.001) (C) Comparison of nucleotide sequence among the AV3 promoter corresponding regions in various AYVV. The regions of various AYVV isolates, corresponding to the AYVV-NT nts 762–806, were used for multiple sequence analysis by ClustalW. The GI numbers of each sequence are shown on the left. The consensus sequence is listed below the alignment, and the putative −35 and −10 boxes are indicated in the red frames.

The −10 and −35 box sequences [18], [19] are involved in the recognition of promoter regions by the sigma factor during transcription initiation. In the previous study [13], we have identified putative −10 and −35 box sequences in the AV3 promoter regions of AYVV-NT and TLCV, and showed that AYVV-NT AV3 promoter exhibited higher activity compared to that of TLCV, possibly due to the higher similarity between the AYVV-NT AV3 promoter core sequences and the canonical −10/−35 box consensus sequences. To examine the functional requirements of the putative −10, −35 boxes in AV3 promoter and to test if the promoter activity were actually related to the sequence similarities, we mutated the putative −10 and −35 boxes in AV3 promoter regions of AVVV-NT to generate a loss-of-function mutant, AY-M, by introducing higher GC contents (Fig. 1A). Since the position of AV3 promoter overlaps the CP ORF, mutants were designed not to alter the amino acids in CP, thus severely limiting the number of mutants allowed. Conversely, we mutated several nucleotides of the TLCV AV3 promoter to the respective ones of AYVV-NT AV3 promoter, in an attempt to create a gain-of-function mutant, TL-M (Fig. 1A), and tested whether the AYVV-NT-like sequences are sufficient to confer higher activity in TLCV AV3 promoter. The promoter strengths of each constructs were then assayed and indirectly indicated by the GFP synthesis rate per cell [16]. Promoter activity assays (Fig. 1B) revealed that the strength of AV3 promoter of AY-M was reduced dramatically to background fluorescence level, indicating a strong requirement of the conservation of −10 and −35 boxes. Unexpectedly, the activity of the TL-M was also reduced even though the −10/−35 box sequences resembled those of AYVV-NT AV3 promoter. The above results indicated that the AV3 promoters indeed harbor the consensus −10/−35 boxes, which are necessary but not sufficient for high promoter activity in *E. coli*. Nucleotides other than those in the −10/−35 boxes are also involved in their promoter activity.

To further test the conservation of the AV3 promoter regions among different isolates and strains of AYVV from different geographical distributions, the corresponding AV3 promoter regions of 20 representative AYVV isolates were aligned using ClustalW [20]. The alignment (Fig. 1C) revealed that AV3 promoter region and the core −10/−35 boxes are highly conserved among different AYVV isolates.

### AV3 promoter can interacted with SSB and sigma 70 of *E. coli*

**Figure 2:**
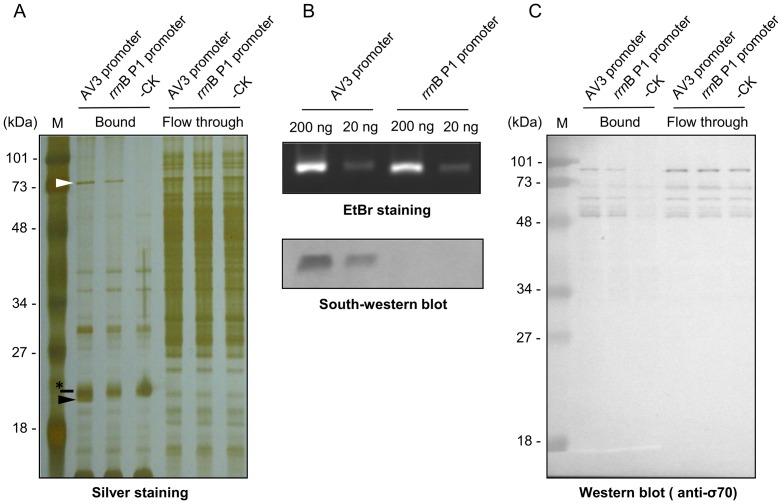
Identification of putative transcription factors interacting with the AV3 promoter. (A) A silver-stained gel showing the products of DNA pull-down assay. The relative molecular masses of the size makers (land M) are shown on the left. The white and black arrowheads indicate the 75 kDa and 20 kDa proteins, respectively, which were further analyzed by MADI-TOF shown in Table 2. The asterisk indicates a non-specific protein present in all three preparations. (B) Demonstration of specific interaction by South-western blot analysis. Different amounts of PCR fragments representing the AV3 and the *rrn*B P1 promoters were electrophoresed through a 1% agarose gel (upper panel), transferred to NC membrane, and probed with His_6_-tagged SSB, followed by detection with His-tag-specific antibody (lower panel). (C) Western blot analysis using monoclonal antibody against Sigma 70.

Previously, we have demonstrated that the AV3 promoter is constitutively active in *E. coli* [13], indicating that the AV3 promoter region should have the ability to interact with certain basal transcriptional factor(s) of *E. coli*. To identify the host factors recruited by AV3 promoter, a biotin-labelled double-stranded DNA fragment of AV3 promoter region was synthesized and used as the bait. The *E. coli rrn*B P1 promoter, which is a strong sigma 70-dependent promoter [21], was used as a control. When compared with the negative control (buffer-only treatment), both the AV3 and *rrn*B P1 promoters bind to a 75 kDa protein (Fig. 2A, white arrowhead), but only the AV3 promoter recruited a 20 kDa protein (Fig. 2B, black arrowhead). Note that a protein (indicated by the asterisk) that migrated closely to the 20 kDa protein was a non-specific protein present in all three preparations (AV3- and *rrn*B P1-bound, and negative control). The results of MALDI-TOF MS analyses (Table 2) showed that the 75 kDa protein is a member of the exonuclease protein family. It is reasonable that both AV3 and *rrn*B P1 promoters interact with one of the exonucleases in *E. coli*, since the baits used in this study are linear double-stranded products of PCR, which should be considered as abnormal in living cells [22], and might be recognized for degradation.

**Table 2:** Identification of the host proteins interacting with AV3 promoter in *E. coli* by MALDI-TOF.

No.	Protein name	species	Observed mass (kDa)^a^	Theoretical mass (kDa)^b^	Coverage (%)	GI number
1	exonuclease family protein	*E. coli*	75	92.45	18.5	419266413
2	single-stranded DNA-binding protein	*E. coli*	20	18.99	33	557274221

a Observed mass (kDa) is based on pre-stained marker.

b Theoretical mass (kDa) is calculated from amino acid sequences.

Unexpectedly, the 20 kDa protein only recruited by the AV3 promoter is identified as the SSB protein. To confirm the finding, a His_6_-tagged SSB protein of *E. coli* was over-expressed, and subjected to South-western blot assays. As shown in Fig. 2B, the SSB proteins can specifically bind to the AV3 promoter, but not the *rrn*B P1 promoter, without the involvement of other host proteins, further confirming the result of pull-down assays. This result is unexpected since SSB does not bind well to the double-stranded DNA [23]. However, in the transcription initiation process, the RNA polymerase recognition and binding would lead to the formation of the open complex structure for the loading of other transcriptional factors [24], which may also need the SSB proteins to maintain the temporarily single-stranded structure. Although we cannot completely rule out the possibility that PCR fragments representing AV3 promoter might form partially single-stranded structures during the assays, the consistency of the electrophoretic mobility of observed bands in EtBr-stained gel and the lack of interaction signal between the SSB proteins and the *rrn*B P1 promoter (Fig. 2B) suggested that the interactions between *E. coli* SSB proteins and AV3 promoter is specific and authentic.

The pull-down assay did not identify the known initiation factors, such as sigma factors, involved in prokaryotic transcription, even in the presence of rifampicin to halt the extension of RNA polymerase initiation complex [17]. Since the AV3 promoter is constitutively active [13] without the involvement of inducers, other environmental cues, or stresses required by other sigma factors, such as sigma 54, 38 or 32 [25]–[27], we therefore directly verified whether the AV3 promoter could be recognized by sigma 70, the basal and most common sigma factor for most genes in *E. coli* [28], [29]. The result revealed that both the AV3 and the *rrn*B P1 promoter could be recognized by sigma 70 (Fig. 2C). The signal intensity detected in the AV3 promoter-bound fraction was equivalent as that in the *rrn*B P1 promoter-bound fraction. Little or no signals of sigma 70 were detected in the negative control. Taken together, these results suggested that the AV3 promoter could at least utilize sigma 70 as their transcriptional initiation factor, and the SSB proteins might also participate in the transcriptional process involving AV3 promoter. However, the possible involvement of other sigma factors was not ruled out.

### AV3 promoter activity is affected by certain AYVV-NT-encoded proteins

**Figure 3:**
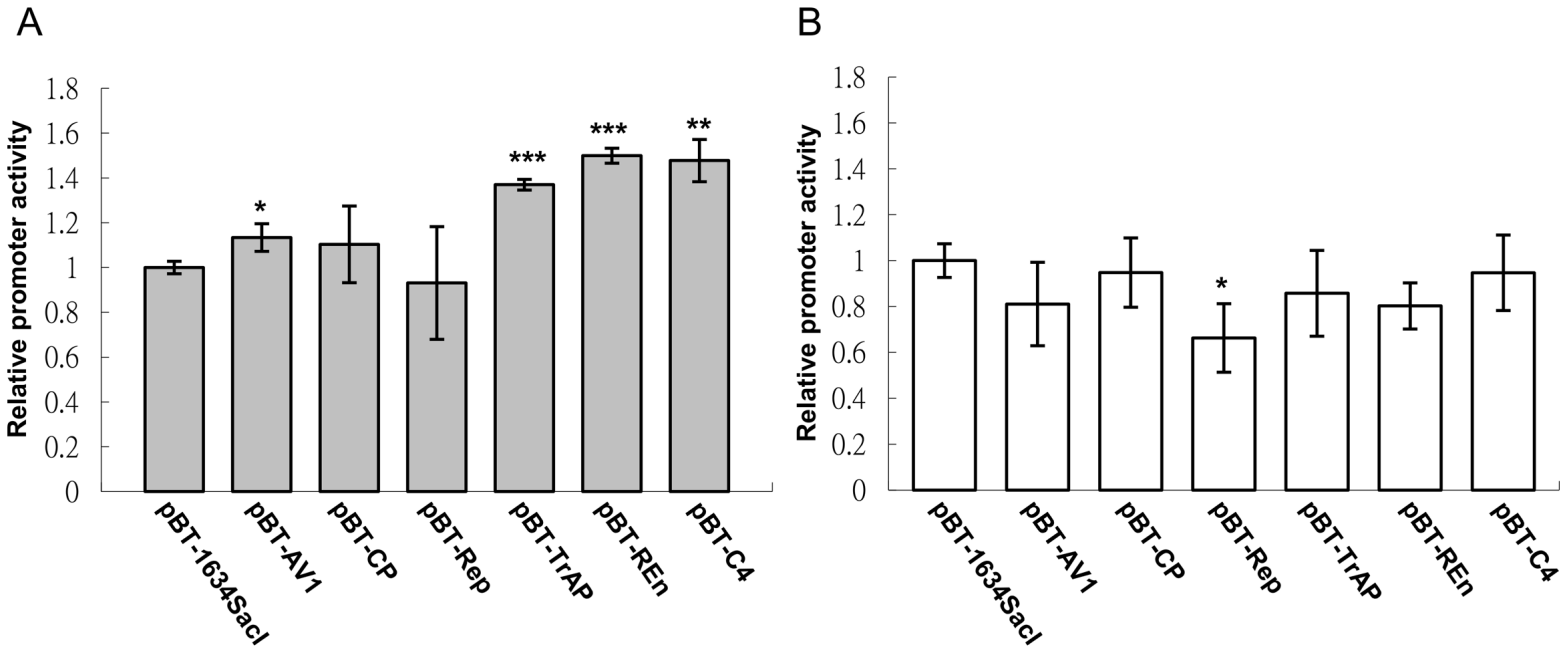
Determination of effects of virus-encoded protein on AV3 promoter activity. Individual viral protein expression plasmids, as indicated under each bar, were co-transformed with the AV3 promoter-driven GFP reporter vector, pAY-WT (A) or the *rrn*B P1 promoter-driven GFP reporter vector, p*rrn*B-P1 (B). The promoter activities were expressed as fold-changes relative to the promoter activity of cells harboring the plasmid pBT-1634SacI, which expresses a lambda-cI protein as a negative control. Asterisks indicate samples that are statistically different from the pBT-1634SacI vector (*, *p*<0.05; **, *p*<0.01; ***, *p*<0.001), as determined by Student's *t*-test.

Previous studies have shown that some geminivirus-encoded proteins could regulate the transcription activity of itself or the other viral genes in plants [30]–[32]. To further understand the influences of AYVV-NT-encoded proteins on the activity of AV3 promoter in the prokaryotic system, the promoter activity assays were performed with the co-expression of individual AYVV-NT-encoded proteins. The AV3 promoter activity in cells co-expressing the lambda-cI protein from the vector pBT-1634SacI was used as a control and served as the basal line for comparison. The result revealed that AV1 (putative movement protein), TrAP (transcription activator protein), REn (replication enhancer) and C4 proteins (pathogenicity determinant) could moderately enhance the AV3 promoter activities to about 1.13 to 1.49 fold compared with negative control pBT-1634SacI (Fig. 3A); whereas the CP and Rep did not significantly influence the expression of GFP driven by AV3 promoter. In contrast, the *rrn*B P1 promoter activity was not significantly affected by the expression of most of the AYVV-NT-encoded proteins, while the Rep protein reduced its activity (Fig. 3B). Although the backbone of the viral protein expression vector harbors a low-copy number *ori*, p15A, which might result in the low expression levels of viral proteins, the amounts of certain viral proteins were enough to exert statistically significant influences on the activities of AV3 promoter. These results suggested that geminivirus AV3 promoter not only can utilize some host factors, but also can be regulated by virus-encoded proteins in *E. coli*.

### The transcription start sites (TSSs) of AV3 promoter in *E. coli* are mapped to nt 803 or 805 of AYVV-NT genome

**Figure 4:**
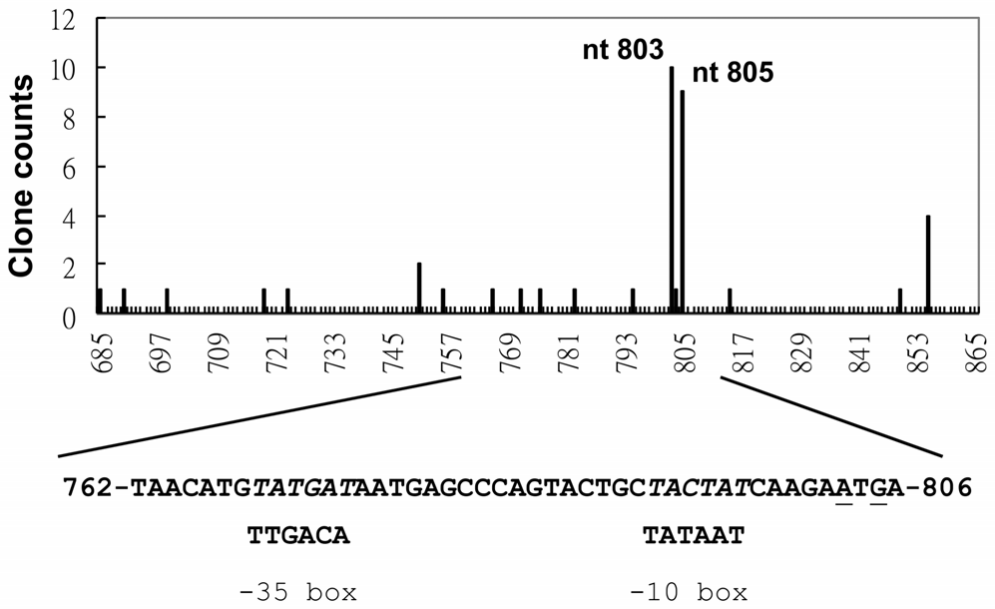
Mapping of transcription start sites (TSSs) of transcripts driven by AV3 promoter in *E. coli*. Mapping of the TSSs downstream to the AV3 promoter in *E. coli* harboring pAY1-7. The numbers of clones with specific 5′-terminus are shown in the chart. The nucleotide positions in AYVV-NT genome are indicated on the X-axis, and clone counts on the Y-axis. The sequence of the AYVV-NT nts 762–806 is shown at the bottom, with the −35 and the −10 region in italics, and the TSSs underlined.

To further characterize the transcription process of AV3 promoter, we mapped the TSSs of AV3 promoter in *E. coli*. An infectious clone of AYVV-NT, pAY1-7, which harbors tandem dimer of the AYVV-NT genome, was used in this analysis. Following 5′ RACE and sequence analysis, we found a group of transcripts harboring 5′ ends at nt 803 or nt 805 (Fig. 4), among 64 independent clones screened. The TSSs of these transcripts were consistent with the putative TSS of canonical prokaryotic promoters at +1 position [33], [34]. Surprisingly, we did not find any longer transcripts suitable for the translation of AV1 or CP in *E. coli*, possibly due to the instability of the longer transcripts. The large proportion of the transcripts with 5′ ends at nt 803 or nt 805 suggested that these transcripts might be the transcription products driven by AV3 promoter in *E. coli*.

### AV3 promoter is likely active in plants, with a putative TSS at nt 856 of AYVV-NT genome

**Figure 5:**
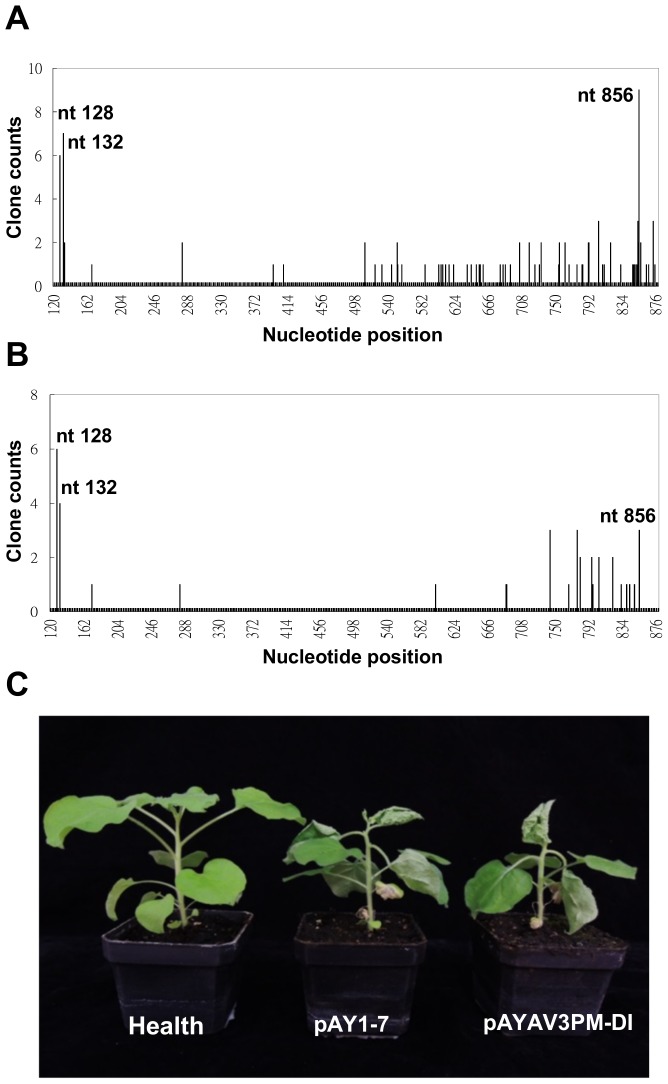
Testing of activity of AV3 promoter in *N. benthamiana* by 5′ RACE and *Agrobacterium*-mediated inoculations. *N. benthamiana* agro-infiltrated with infectious clones of wild type AYVV-NT, pAY1-7 (A), or mutant pAYAV3PM-DI (B), were used for mapping of the 5′-ends of virion-sense mRNA transcripts by 5′ RACE. The numbers of clones with specific 5′-terminus are shown in the bar chart. X-axis, the nucleotide positions in AYVV-NT genome; Y-axis, clone counts. (C) Symptoms induced by wild type and mutant AYVV-NT. Whole plant image of the healthy and inoculated *N. benthamiana* plants infiltrated with *A. tumefaciens* harboring AYVV-NT wild type (pAY1-7) and mutants (pAYAV3PM-DI) at 21 dpi is shown.

To test whether AV3 promoter is active also in plants, we mapped the TSSs of the virion-sense transcripts in the AYVV-NT infected *N. benthamiana*. We sequenced more than 100 independent clones and found that there are two major groups of virion-sense transcripts. The first group comprises the transcripts with 5′ end at nt 128 or nt 132 of AYVV-NT genome, which are consistent with the mRNAs for AV1 and CP reported previously for other begomoviruses [35]. The second group comprises the transcripts with 5′ terminus at nt 856, which likely represent the transcripts driven by the AV3 promoter (Fig. 5A). We also examined the 3′ terminus of the transcripts, and found that the majority of the transcripts have the 3′ terminus at nts 1081–1084.

It has been well-known that *cis*-elements required by the prokaryotic and eukaryotic promoter are different: the prokaryotic promoters harbor the consensus −35 and −10 boxes for RNA polymerase recognition, while the eukaryotic promoter may contain the TATA box, TFIIB recognition element (BRE), initiator element (Inr), and downstream promoter element (DPE) [36], [37]. Thus, it is reasonable that the transcripts obtained from prokaryotes and eukaryotes may have different 5′ends. Although the precise positions of these eukaryotic *cis*-elements in AYVV-NT AV3 promoter remain undefined, the activity of AV3 promoter in bacteria and plants was supported by the presence of these transcripts with proper 5′ termini.

Computer assisted predictions were used to search for putative eukaryotic *cis*-elements in the AV3 promoter region. The result of predictions by SCOPE [38] revealed several overlapping candidates of eukaryotic *cis*-elements in AV3 promoter, located near nts 785–801 of AYVV-NT genome, which also overlap with the prokaryotic conserved −10 box (nts 792–797). As shown in Fig. 1B, mutations in the core elements of AV3 promoter effectively abolished the AV3 promoter activity of AYVV-NT in *E. coli*. We thus tried to test the influence of these prokaryotic *cis*-elements on virus infection cycle in plants. We generated an infectious mutant, pAYAV3PM-DI, containing the mutated AV3 promoter as shown in Fig. 1A (AY-M), and verified the TSSs of the transcripts driven by the mutated AV3 promoter (Fig. 5B). Among the 38 clones sequenced, the virion-sense mRNAs with TSSs at nt 128 or nt 132 remained to be the majority. In contrast, the number of transcripts with TSSs at nt 856 were reduced (Fig. 5B), compared with that from wild type-infected plants (Fig. 5A). Although the possibility that the mutated nucleotides affected the expression of CP and the stability of respective mRNA could not be precluded, the decrease of transcripts with TSS at nt 856 relative to those with TSSs at nt 128 or 132 suggested that the mutations in the −10 box might interfere with, but did not completely abolish, the activity of AV3 promoter in plants. This result also provided support for the presence of eukaryotic *cis*-element in the AV3 promoter region in close proximity to, or overlapping, the prokaryotic core motifs.

The symptoms of *N. benthamiana* induced by the wild type and the mutant AYVV-NT at 21 dpi were shown in Fig. 5C. No significant differences in symptom or infectivity were observed. The result of inoculation assays suggested that AV3 promoter might not be involved in agrobacterium-mediated infection processes, or that the interference on AV3 promoter activity introduced by the mutations might not be enough to cause distinguishable difference in symptom expression. The agrobacterium mediated infectivity assay on *N. benthamiana* is an artificial infection process. Begomoviruses are transmitted in a persistent manner by whitefly vectors [39], which harbor prokaryotic symbionts in the digestive system. Whether the AV3 promoter is involved in the natural infection cycle on the native host plant (*Ageratum spp.*) remains to be elucidated.

### AV3 promoter is functional in yeast and has a short translatable ORF

**Figure 6:**
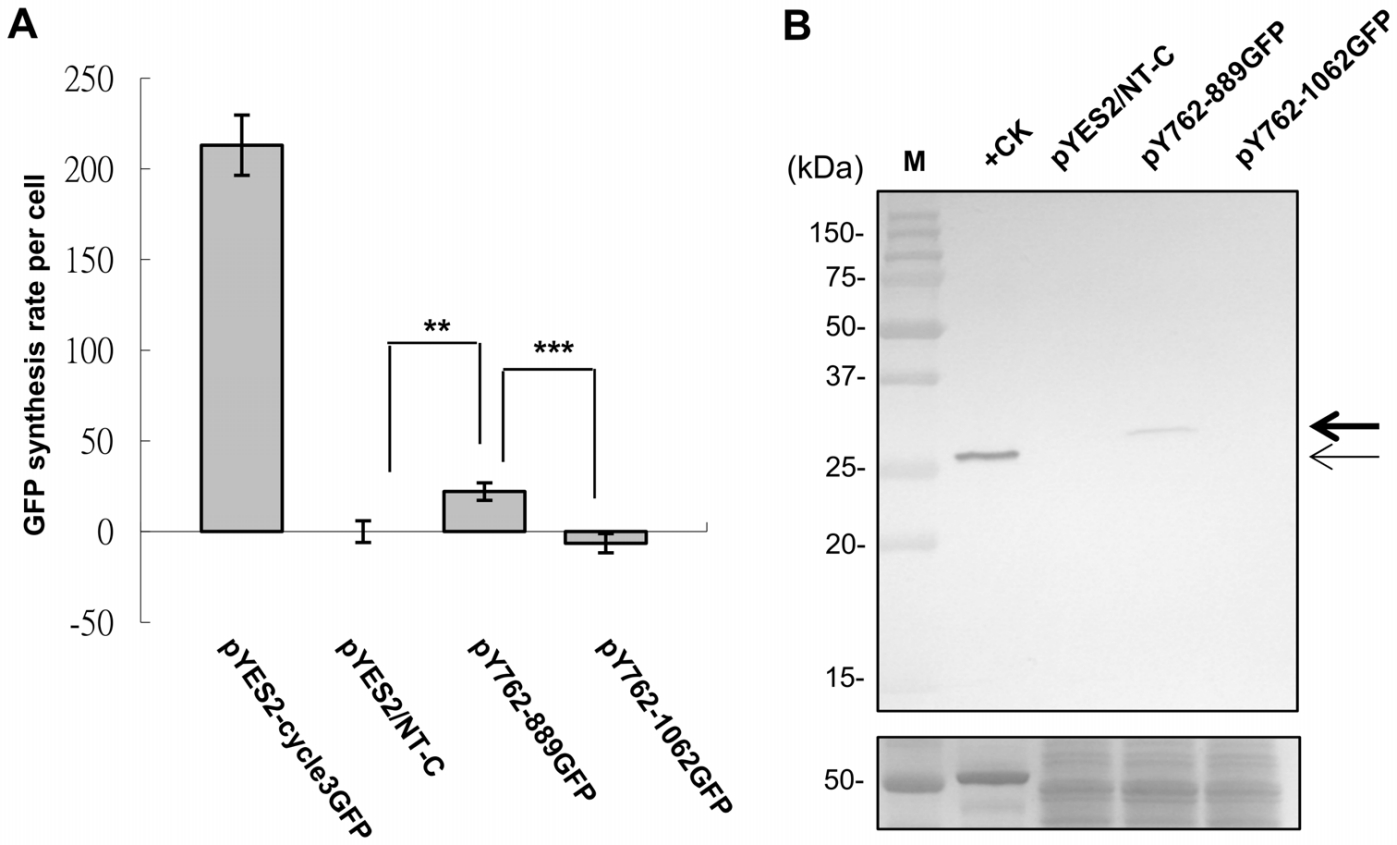
Analysis of the AV3 promoter activity in yeast (*S. cerevisiae*). (A) The OD_600_ and GFP fluorescence of cultures containing the indicated constructs were measured as described above, except that the readings were taken at 0 h and 4 h to accommodate for the slower growth rate of the yeasts. The empty vector pYES2/NT-C was used as a negative control. Asterisks indicate value-pairs that are statistically different (**, *p*<0.01; ***, *p*<0.001), as determined by Student's *t*-test. (B) Western blot analysis to verify the translatability of ORFs downstream to AV3 promoter in yeast cells using GFP-specific monoclonal antibody (upper panel). The transiently expressed GFP in *N. benthamiana* was used as a positive control and size marker (+CK). The positions of control and fused GFP are indicated by the thin and thick arrow, respectively. The coomassie blue-staining gel (lower panel) is shown as a loading control.

In the previous study, we have demonstrated that the AV3 promoter could drive the expression of the first short downstream ORF at nts 866–892 in *E. coli* [13]. To further verify the activity of AV3 promoter and the downstream ORF in eukaryotic system, we used similar GFP-fusion reporter strategy in yeast (*Saccharomyces cerevisiae*) cells. The yeast cells do not harbor chloroplasts or other plastids, ruling out the complexity that the AV3 promoter and downstream ORF might be active in these prokaryotic organelles within the eukaryotic cells [40]–[43]. We generated two constructs harboring the same fragments as we created for assays in *E. coli* [13]. These two constructs both contain the AV3 promoter starting from nt 762 of AYVV-NT genome, but with different 3′ ends fused to the N-terminus of the reporter GFP ORF: in pY762-889GFP, the first downstream ORF (nts 866–889 in frame +3 relative to nt1 of AYVV-NT genome) was fused to GFP fragment; whereas in pY762-1062GFP, the second ORF (nts 901–1062 in frame +2) was fused. The results of promoter activity assay showed that the GFP fluorescence intensity was significantly higher for yeast harboring pY762-889GFP, as compared with those harboring pY762-1062GFP (*p*<0.001) and the negative control pYES2/NT-C (*p*<0.01) (Fig. 6A). To verify the expression of GFP driven by AV3 promoter in yeast, a western blot analysis using GFP specific antibody was performed. As shown in Fig. 6B, the detection of the fused GFP with increased molecular weight (indicated by the thick arrow) in yeast cells harboring the plasmid pY762-889GFP confirmed that the AV3 promoter is active in yeast and that the first ORF downstream to AV3 promoter is translated, which increased the size of the fused GFP as compared to the control GFP expressed in *N. benthamiana* (indicated by the thin arrow).

## Discussion

In this study, the features related to the regulation and activities of AYVV-NT AV3 promoter were further characterized in both prokaryotic and eukaryotic environments. The *cis*- and/or *trans*-elements involved in AV3 promoter-driven transcription, and the TSSs were characterized in bacteria and plants. The expression of the first downstream ORF was also demonstrated in yeasts. These results hinted that AV3 promoter might still retain certain degrees of activity in the present-day infection cycles of certain begomoviruses.

The interaction between AV3 promoter and *E. coli* SSB protein is intriguing and might shed some light on the evolutionary history of geminiviruses. SSB proteins are mainly involved in the replication, repair, and recombination processes of prokaryotic DNAs [44]. For transcription initiation, SSB proteins are usually not required. However, SSB proteins are known to be involved in the transcription of certain bacteriophages. It has been shown that the *E. coli* SSB can activate the transcription of supercoiled double-stranded DNA of phage N4 by the virion RNA polymerase [45], [46]. The *E. coli* SSB may mediate the recycling of DNA templates during the transcription by phage N4 virion RNA polymerase [47]. Phage N4 also encodes a viral SSB for the expression of late genes in the infection cycle [48]. Our observations hinted that geminiviruses might share similar evolutionary origins with certain bacteriophages. Although the underlying mechanism for transcription might be different for phage N4 and geminiviruses, it is possible that geminiviruses might retain the ability to recruit SSB protein in the transcription processes during the evolutionary history.

On the other hand, the interaction between AV3 promoter and SSB might also be involved in the replication process of AYVV genome, since some DNA viruses encode SSB proteins on their genomes [49]–[52], and the eukaryotes also encode SSB proteins for processes involving DNA synthesis [53], [54]. The actual function of the interactions between SSB and AV3 promoter region require further investigation.

The mutations in the −10 box region of AV3 promoter also reduced the activity of AV3 promoter in *N. benthamiana* (Fig. 5B), suggesting that the *cis*-elements required in prokaryotic and eukaryotic systems either overlap or exist in close proximity. However, the differences in TSSs mapped for transcripts driven by AV3 promoter in bacteria and plants (Fig. 4 and 5) indicated that different transcription initiation mechanisms are involved. Furthermore, the TSS of AV3 promoter-driven transcripts in plants was mapped to nt 856, which is only 9 nucleotides away from the downstream ORF (starting at nt 866). It has been proposed that at least 7 nucleotides are required as the proper spacing for efficient translation [55]. However, for geminiviruses, the close proximity between the TSS and first codon of downstream ORF is known, and might be involved in the regulation of gene expression [35].

Although the activity of AV3 promoter and the translatability of the downstream ORF are shown in yeast cells here, the actual biological functions require further explorations. The nucleotide sequences of AV3 promoter region are highly conserved among different isolates of AYVV from different geographical distributions (Fig. 1C) over the long evolutionary history. Although it is possible that the conservation of the sequences might result from the highly conserved CP genes of begomoviruses [56], the high nucleotide sequence identities in AV3 promoter regions shared among different AYVV species suggest that AV3 promoter might be functional in the original host plants, *Ageratum spp.*, since different AYVV strains maintained highly similar nucleotide sequences to code for the same amino acids, instead of allowing other synonymous mutations. One of the functions of AV3 promoter is the involvement in interactions with the endo-symbiotic bacteria in the whiteflies, which might be important for insect transmission. Further experiments are needed to verify the function of AV3 promoter in *Ageratum* plants and/or whitefly vectors. The AV3 promoter and the downstream ORF might be a remnant of evolution, or possess actual biological function(s) that remain to be elucidated.

In conclusion, the results presented in this study confirmed the activity of AV3 promoter in a prokaryotic system, and suggested that AV3 promoter might possess a low level of activity in certain eukaryotic systems. The observations revealed more details of a cryptic promoter residing in the genome of AYVV, and provided further hints to the evolutionary history of geminiviruses.
